# A Multidisciplinary Approach toward High Throughput Label-Free Cytotoxicity Monitoring of Superparamagnetic Iron Oxide Nanoparticles

**DOI:** 10.3390/bioengineering6020052

**Published:** 2019-06-10

**Authors:** Sonia Abad Tan, Georg Zoidl, Ebrahim Ghafar-Zadeh

**Affiliations:** 1 Biologically Inspired Sensors and Actuators Laboratory, Lassonde School of Engineering, York University, Ontario, Toronto, ON M3J 1P3, Canada; sat1276@yorku.ca; 2 Department of Biology, York University, Toronto, ON M3J 1P3, Canada; gzoidl@yorku.ca; 3 Department of Psychology, York University, Toronto, ON M3J 1P3, Canada; 4 Department of Electrical Engineering and Computer Science, York University, Toronto, ON M3J 1P3, Canada

**Keywords:** SPIONs, high-throughput, viability, neuroblastoma 2A, impedance-based assay

## Abstract

This paper focuses on cytotoxicity examination of superparamagnetic iron oxide nanoparticles (SPIONs) using different methods, including impedance spectroscopy. Recent advances of SPIONs for clinical and research applications have triggered the need to understand their effects in cells. Despite the great advances in adapting various biological and chemical methods to assess in-vitro toxicity of SPIONs, less attention has been paid on the development of a high throughput label-free screening platform to study the interaction between the cells and nanoparticles including SPIONs. In this paper, we have taken the first step toward this goal by proposing a label-free impedimetric method for monitoring living cells treated with SPIONs. We demonstrate the effect of SPIONs on the adhesion, growth, proliferation, and viability of neuroblastoma 2A (N2a) cells using impedance spectroscopy as a label-free method, along with other standard microscopic and cell viability testing methods as control methods. Our results have shown a decreased viability of the cells as the concentration of SPIONs increases with percentages of 59%, 47%, and 40% for 100 µg/mL (C4), 200 µg/mL (C5), 300 µg/mL (C6), respectively. Although all SPIONs concentrations have allowed the growth of cells within 72 h, C4, C5, and C6 showed slower growth compared to the control (C1). The growth and proliferation of N2a cells are faster in the absence or low concentration of SPIONS. The percent coefficient of variation (% CV) was used to compare cell concentrations obtained by TBDE assay and a Scepter cell counter. Results also showed that the lower the SPIONs concentration, the lower the impedance is expected to be in the sensing electrodes without the cells. Meanwhile, the variation of surface area (∆S) was affected by the concentration of SPIONs. It was observed that the double layer capacitance was almost constant because of the higher attachment of cells, the lower surface area coated by SPIONs. In conclusion, impedance changes of electrodes exposed to the mixture of cells and SPIONs offer a wide dynamic range (>1 MΩ using Electric Cell-substrate Impedance electrodes) suitable for cytotoxicity studies. Based on impedance based, viability testing and microscopic methods’ results, SPIONs concentrations higher than 100 ug/mL and 300 ug/mL cause minor and major effects, respectively. We propose that a high throughput impedance-based label-free platform provides great advantages for studying SPIONs in a cell-based context, opening a window of opportunity to design and test the next generation of SPIONs with reduced toxicity for biomedical or medical applications.

## 1. Introduction

Superparamagnetic Iron Oxide Nanoparticles (SPIONs) have attracted the attention of researchers for clinical and research purposes due to their structural and magnetic properties that make them suitable for drug delivery, disease diagnostics and treatment purposes [1,2]. SPIONs are chemically made up of magnetite (Fe_3_O_4_) and maghemite (γ-Fe_2_O_3_) [3]. By applying a magnetic field, SPIONs are directed as nanoscale carriers to a target organ in the body. For instance, several studies have shown that SPIONs can cross the Blood Brain Barrier (BBB) [4,5] and deliver the drug into the brain [6,7]. In these studies, the uptake of SPIONs by the astrocytes [8] can be used as an indicator of nanoparticle (NP) delivery through BBB. Other studies have shown that SPIONs lower than a certain concentration level are not toxic compared to other higher saturation magnetic NPs [9,10,11]. The Food and Drug Administration’s (FDA) approval [12] of SPIONs as MRI contrast agents has created an intense interest in promoting the use of these nanomaterials in humans [13] for various clinical applications, including diagnostic and treatment of brain diseases over the last decade [14,15]. 

Despite significant advances of SPIONs for various life science applications, many research studies should still be conducted to enhance our understanding of the effects of SPIONs with different concentrations on cellular activities. It is in this direction that this paper progresses, specifically by focusing on the interaction of SPIONs and brain cells using various methods, including impedance spectroscopy. 

**Figure 1:**
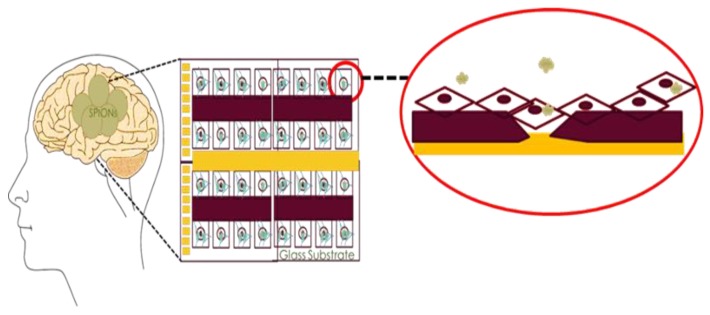
Schematic representation of the interaction of the brain cells with SPIONs when studied in in-vitro using an impedance-based assay.

Figure 1 illustrates the proposed in-vitro method in this paper to mimic the uptake of SPIONs on brain cells. This method offers great advantages for studying the interaction of SPIONs and the brain cells as described and demonstrated in the next sections. The remainder of this section provides a comprehensive review of the literature to explain the advantages of SPIONs for neuronal studies, and other applications in Section 1.1, followed up by Section 1.2 that briefly reviews the in-vitro studies of the effects of NPs on cells. 

### 1.1. SPIONs Applications

SPIONs have demonstrated great advantages for various life science applications including non-invasive Magnetic Resonance Imaging (MRI) [11], diagnosis of ailment, drug delivery and development [16], thermotherapy [17], biological separation [17], cell transfection [18], immunoassays [19], gene delivery [20], tissue engineering [21], and cell tracking in cancer and its treatments [22]. Some important SPIONs applications are briefly put forward as follows.

- MRI Contrast Agent: MRI is used to visualize and track a diseased portion of the brain. The strength of the signal is influenced by the two-relaxation times of water protons, the longitudinal (T_L_) and transverse (T_T_) [23,24]. For the image refinement, contrast agents are utilized to decrease T_L_ and T_T_ relaxation times. The SPIONs act as negative contrast agents, producing a negative signal on T_T_ weighted images and enhancing T_T_ contrast [25].
- Tumor Diagnostics and Therapy: Functionalized SPIONs can play an essential role in the delivery of therapeutic components and subsequently for initiating tumor cell death [26]. A biocompatible coating on SPIONs provides suitable functional groups for conjugating with tumor cells [27,28]. For instance, SPIONs can be attached to the anti-IL-1*β* monoclonal antibody to be used for MRI diagnoses and targeted therapy by neutralizing IL-1*β* which is overexpressed in the epileptogenic area of an acute rat model with temporal lobe epilepsy [29], a disease in the brain associated with inflammation [30].
- Thermotherapy: To implement a hyperthermia treatment, SPIONs can be introduced in the body through a magnetic delivery system or a local injection to the affected area [31]. SPIONs can vibrate and produce heat in an interchanging magnetic field [8,9]. The generated heat can be used for thermotherapy purposes.
- Crossing BBB: As previously mentioned, recent studies have reported that SPIONs can enter the brain without causing damage to the blood-brain barrier [32]. To date, many types of research have been conducted to understand the BBB mechanisms and enhance the BBB permeability using functionalized SPIONs. Among these efforts is an optimized in-vitro BBB model, which was recently being reported using mouse brain endothelial cells and astrocytes [33,34]. Also, experimental data demonstrated how one could modify SPIONs to deliver drugs to the brain to more effectively treat a wide range of neurological disorders [35].
- Drug Delivery: SPIONs are widely used because of their larger surface to mass ratio [36] compared to other NPs, their quantum properties [37] and their ability to absorb [38] and carry other compounds. The aims for such NP entrapment of drugs are either enhanced delivery to or uptake by, target cells and a reduction in the toxicity of the free drug to non-target organs. Both situations will increase the ratio between the doses resulting in therapeutic efficacy and toxicity to other organ systems. For these reasons, the creation of long-lived and target-specific NPs and accurate toxicity studies should be performed to increase the advantages of these particles for the applications mentioned earlier [10]. It is noteworthy that SPIONs are not stable under physiological conditions due to the reduction of electrostatic repulsion, which causes NP aggregation. To re-disperse SPIONs in biological media, further surface modifications are applied in particular on the commercially available SPIONs [39].

### 1.2. Effects of NPs on Cells: In-Vitro Studies

To date, many papers have reported the advantage of NPs for drug delivery purposes using in-vivo animal models [40,41]. In comparison with in-vivo studies of NPs, less attention has been paid to studying the effect of NPs using in-vitro cell culture models. In general, even though in-vivo animal model studies offer exceptional advantages for testing NPs or other drugs in human-like fully functional organs, in-vitro cell culture models can also provide unique benefits for various fundamental biological and clinical studies. These advantages include higher environmental control, less variability, low complexity and higher repeatability [42]. It is noteworthy to mention that N2a cells have been used widely for in-vitro neuroscience studies due to their capacity to differentiate [43] and respond to electrophysiological stimulation [44]. In this paper, N2a cells were used as an in-vitro cell culture model. 

#### 1.2.1. Fundamental Effects

In in-vitro models, NPs including SPIONs can directly be added to the cell culture, and they interact with the culture medium [45], aggregate in the intercellular spaces, attach to the cell membrane [46] and affect intracellular parts of the cell [47]. Indeed, the culture medium can change the properties of NPs by forming a protein coat covering the entire NP [48]. This may increase the adhesion properties of NPs for the attachment to the cell membrane. NPs’ distinctive physicochemical properties with increased responsiveness and propensity to pass through the cell membrane and other biological barriers cause stress and induce cytotoxicity [49]. Herein, the major effects of NPs on cells are highlighted.

- Effect on cell membrane: All types of NPs including SPIONs can be assimilated into the cell via different processes and all these types passe through the protective barrier of the cell—the cell membrane. As NPs make their way through the cell membrane, they affect the major components of the membrane, the proteins [50,51] and the lipid bilayer [52].
- Effect on Lysosomes: A study using silica (SiO_2_) NPs on human cervix carcinoma (HeLa) cells, had shown that NPs disrupt normal activities of the lysosomes by causing damage in their cargo delivery via autophagosomes. Although the autophagy-mediated protein turnover and degradation of internalized epidermal growth factor were affected, this did not induce cell death [53].
- Effect on cytoplasmic organelles: Experimental investigation has shown evidence that NPs affect cytoplasmic organelles like the mitochondria [54] and nucleus [55]. Another study had shown that even if using gold nanoparticles (GNPs) does not cause accumulation within the mitochondria, NPs close to the organelle could still enhance damage due to the delocalization of photoelectrons from the cytosol. Furthermore, the presence of GNPs in the cytosol increases the energy deposition in the mitochondrial volume more than the presence of GNPs within the nuclear volume [56].
- Effects on the cell activities: The effect of GNPs on cell differentiation and maturation has been highlighted in another study. It has been observed that the cells developed longer neuronal outgrowth in the presence of GNPs [57,58].
- Other effects: The exposure of the cell to NPs brings about harmful effects such as damage mitochondrial function, inflammation, the formation of apoptotic bodies, membrane leakage of lactate dehydrogenase, reactive oxygen species (ROS) production, increase in micronuclei, and chromosome condensation [49]. In such cytotoxicity studies, there are various indicators such as micronuclei that are an indicator of gross chromosomal damage that is used to measure genotoxicity.

Despite significant advances in the studying of NPs in different types of cells, still, the effects of many kinds of NPs on various parts of cells or different types of cells have not been studied. In this paper, we only focus on exploring the effects of SPIONs on N2a using three cellular level indicators, namely; cell viability, morphology, and cell adhesion. 

#### 1.2.2. In-vitro Toxicity Assays

As previously mentioned, NPs can affect many different parts of the cell. Thus, various conventional toxicity assays are required to measure the damage caused by NPs. As per the literature, these assays include 3-(4,5-Dimethylthiazol-2-yl)-2,5-diphenyltetrazolium bromidefor (MTT) assay, cell metabolic activity assay (WST-1), Cell Proliferation 5-Bromo-2´-Deoxyuridine (BrdU) Analysis [59,60], lactate dehydrogenase (LDH) leakage, fluorescent propidium iodide (PI), [3H] thymidine, Clonogenic assays, Electron paramagnetic resonance (EPR), Lipid peroxidation Assay, Enzyme-linked immunosorbent assay (ELISA) and Trypan blue dye exclusion (TBDE) test [61]. 

Most recently, various new sensing methods are being used in toxicity studies. Among these new methods, Fritzsche et al. reported a cell-based fluorometric sensor system [62,63]. This system uses fluorescence data, which is generated by connecting a multi-well culture plate to a fluorescence spectrometer. With software, concentration curves were further analyzed. These curves are also used to indicate the concentration of the toxicant. In another effort, impedance spectroscopy [64,65] has opened the possibility of a faster, real-time, high-throughput acquisition of results. For instance, an Electric cell-substrate impedance sensing (ECIS) device that was used to monitor mammalian cell activities under the presence of toxicant. This device was connected to a PDMS perfusion system and impedance spectroscopy system [66]. Also, the impedance-based method was used to investigate the change in the activity of macrophage cell line J774, epithelial cell line MDCK and fibroblasts [67]. In this direction, another effort was made by Zhu et al., who presented the lateral flow immunoassay (LFIA) to determine toxicity at the genetic level [68]. In another attempt, a multichannel dissolved oxygen sensor consisting of a 96-well electrodes biosensor was introduced by the group of Sadik et al. to detect toxicity. Their measurement setup was used for monitoring the amount of oxygen used by the cell [69]. Also, the paper authored by Özel et al. provided information on using electrochemical approaches in monitoring the effect of NPs [70]. 

The first section presents a comprehensive review of several related papers of literature on the different SPIONs applications and effects of NPs on cells in-vitro studies. The next section emphasizes related works on SPIONs’ cytotoxicity studies and high throughput impedance-based cellular analysis. The third section gives a clear understanding of the materials used in this study and explains the methods that had been performed to gather the data. Section 4 displays the results in the form of graphs, figures, and tables. Each result is explicitly discussed, including the integration impedance spectroscopy results and equivalent circuit model. Further discussion can be seen in Section 5 where economical and time assessments as well as a high throughput analysis device for the future were highlighted. Finally, the last section establishes conclusions based on the results.

## 2. Related Works

As the focus of this paper is placed on iron oxide NPs, in this subsection, a more comprehensive review of related works is provided. 

### 2.1. SPIONs’ Cytotoxicity Studies

To date, several studies have examined the cytotoxic potential of magnetic nanoparticles (MNPs) or SPIONs by employing a range of different surface coatings. In this subsection, we review the toxicity effect of NPs, particularly SPIONs on living cells, as seen in Table 1. This table shows various in-vitro toxicity studies using different types of cell lines, NPs, and tools for evaluation purposes. For instance, Marcus et al. [71] reported the effect of uncoated and coated MNPs on Rat pheochromocytoma PC12 cells (R-PC12). As seen in Table 1, uncoated MNPs did not diffuse in the cells and only aggregated on the cell membrane. Two other types of coated MNPs, namely Starch-magnetite MNPs and Dextran-magnetite MNPs showed lower and higher viability (or less toxicity) effects respectively in comparing with uncoated MNPs. In their work, in addition to an MTT viability assay, Electrophysiological and Morphometric methods were used for full cytotoxicity analysis. As shown by Mahmoudi et al. [72] both Polyvinyl alcohol (PVA) coated and uncoated SPIONs manifested a decreased viability of L929 mouse fibroblast cells. This effect was also demonstrated in the MTT assay along with visible ultraviolet spectroscopy and optical microscopy. Moreover, as tested by Jarockyte et al. [73] a second generation tetrazolium dye, Cell Proliferation Kit II (XTT), had been used to assess the viability and proliferation of a mouse embryonic fibroblast (NIH3T3) which manifested a slight decrease of viability within 3–24 h of incubation when uncoated SPIONs were introduced.

In a similar study, Magdolenova et al. employed several other viability assays including trypan blue exclusion, relative growth activity assay, and a Cytokinesis-block proliferation index (CBPI) [74]. For the results, they demonstrated a reduction of viability of TK6 human lymphoblast cells exposed to coated and uncoated SPIONs. As revealed by Ying and Hwang [75] when using a Fluorescein diacetate uptake-based cytotoxicity assay, toxicity varies depending on the concentration and particles of NPs. Meanwhile, another study indicated that bEnd.3 showed reduced viability when exposed to a coated NPs as revealed by an MTT assay [76]. Likewise, an investigation that made use of MTT, TBDE, and a resazurin-based PrestoBlue (PB) assay revealed no death of cells, but proliferation was decreased [77]. In PB assay, the red color can be used as an indicator of A549 viability as a result of the reduction of PrestoBlue to resorufin [78]. It was mentioned by Soenen et al. [79] that modifying NPs’ coatings such as dextran, carboxydextran, lipid, and citrate can also affect adhesion and proliferation but does not affect the surface area of the C17.2 and PC12 cells as revealed by lactate dehydrogenase (LDH) assay and manual cell counting using a Bürker chamber. A similar study [80] using an LDH assay demonstrated a high viability of retinal ganglion cell (RGC) cells exposed to a dimercaptosuccinate (DMSA) NPs. It is also worth mentioning that two separate studies found in reference [81] and reference [82], using SPIONs coated with doxorubicin (DOX) and dimercaptosuccinic acid (DMSA) respectively had exhibited different viability results using MTT and TBDE assays as seen in Table 1. In both studies, MCF-7 cells were incubated with SPIONs. It seems DMSA coated on SPIONs provide enhanced viability in comparison with DOX coated on SPIONs. 

### 2.2. High Throughput Impedance-Based Cellular Analysis

Impedance-based techniques have been widely reported for the assessment of cellular activities such as adhesion of cells [83,84] and cell growth [85]. In these techniques, the cells are cultured on the top of sensing electrodes connected to an impedance spectroscopy system. This system can measure the impedance of the electrode exposed to the biological materials. These techniques have been successfully used to monitor the attachment and growth of *vero* and human cells [86] due to the variation of their electrical properties. For instance, the activation of acetylcholine receptors in N2A results in higher conductivity that could be measured using Impedance based techniques. 

Many efforts have been made to show the advantages of impedance analysis using 2D or 3D cell culture models [87] for various cellular analysis. It has been highlighted in the work of Seriburi and Meldrum (2008) that impedance can be used to monitor adhesion and to spread junctional epidermolysis bullosa gravis (JEBG) cells [88]. In their work, morphological changes such as a change in shape and confluence were accompanied by the change of impedance. Also, in another study, the impedance change was correlated to the number of colon cancer cells in culture [89]. Moreover, an impedance-based Electric Cell-substrate Impedance Sensing (ECIS) was used to investigate cell morphology, basal and substratum distance, and capacitance of the cell membrane’s anatomical planes of epithelial cells [90]. This was carried out in order to examine the change of Ca^2+^ concentration in kidney cells. According to Wang et al., the impedance can be used to assess cellular processes in terms of cell death with millisecond time resolution [91].

By considering the high demand and urgency of screening and evaluating the effect of different NPs used in the clinical sector, impedance-based high throughput screening systems (HTSS) is the best solution to meet the challenge. This system offers the advantages of high speed and simultaneous real-time assessments of the different effect of NPs [92]. Despite major advances of impedance-based systems, relatively little attention has been paid to using these systems for toxicity assessment of NPs. Among few studies that have done so, Moe et al. reported a microelectronic sensing device consisting of 96 micro-wells capable of measuring the real-time activities [93]. In their work, an impedance-based method was used for demonstrating various assessment methods related to cellular activities. These methods include the assessment of toxicity level, cell death, assessment of cell membrane integrity, attachment and proliferation. 

As previously mentioned, to date, many papers have reported the advantages of impedance-based and capacitive techniques [94] for monitoring the growth of living cells. However, this project is the first to demonstrate the advantage of an impedance sensing method as the core of a high throughput platform for monitoring the effect of SPIONs on the cells in culture. In this work, as a control of the proposed impedance-based results, we also use other standard biological methods as described in Section 2. 

Table 2 highlights the uses of impedance-based methods in cellular analysis. For instance, in a study conducted by Williams et al. [95], an impedance-based assay was used in monitoring the distribution and reaction of cells using an implanted electrode. Meanwhile, several types of research were performed by Szulcek et al. [96] and Arias et al. [97] using ECIS to observe adhesion, spreading proliferation, and migration of cells; maturation of a confluent cell barrier, wound healing, and apoptosis. Another notable work was conducted by Kuzmanov to study cell integrity and permeability using an impedance technique. Additionally, a change in cell shape can be monitored by the impedance-based device as an effect of chlorotoxin [98]. In another attempt made by Peters et al. [99], cytotoxicity monitoring was performed using an impedance-based assay. 

As per the above discussion, the impedance-based cellular monitoring can be used as a reliable method for cellular analysis. As described in the next chapter, we will use this impedance-based method for the cytotoxicity study of SPIONs. 

Despite the volume of studies conducted for cytotoxicity of SPIONs, it is still one of the pressing challenges in the clinical and biomedical field. In the previous paragraphs, it has been highlighted that a certain amount of SPION may pose a danger and may cause alteration to cellular attributes or behavior. 

## 3. Materials and Methods

This section presents the materials and methods used in the study. It also describes the research process and the details of the methods used in an operational manner.

### 3.1. Materials

#### 3.1.1. Organism

The Neuroblastoma 2a cells (Neuro 2a or N2a cells) are a fast-growing mouse neuroblastoma cell line derived from an albino mouse strain [101]. This cell line was purchased from ATCC®. The maintenance, storage, and manipulation of the cell line used in this study were performed at the Medical Devices Laboratory at the Bergeron Building, York University. 

#### 3.1.2. Chemicals

Most of the chemicals and reagents, including DMEM, FBS, PS, PBS, and trypan blue dye). Ethanol (Commercial ALC.), fetal bovine serum (FBS) (Life Technologies) and water (ultrapure type I) were purchased from Sigma-Aldrich (Oakville, Canada). The spherical shape SPIONs were purchased from Skyspring Nanomaterials, Inc (Houston TX, USA). The average size of SPIONs used in this study was ~10–15 nm. Characterization of SPIONs in terms of XRD pattern, SEM image and magnetic properties is available from reference [102].

##### Solutions and Media for Cell Culture

Subculturing the cell was performed using phosphate buffered saline (PBS) (Sigma-Aldrich Canada Co., Toronto, Canada), containing 0.9% Sodium chloride, 99% Water, 0.0144% Potassium dihydrogenorthophosphate, 0.0795% Sodium monohydrogen phosphate, heptahydrate, pH 7.2, sterile-filtered, trypsin-EDTA (Sigma-Aldrich Canada Co.) containing 0.05% trypsin, 0.02% EDTA (1×) in D-PBS (PAA). Meanwhile, Dulbecco’s Modified Eagle Medium (DMEM), (Sigma Life Science, Darmstadt, Germany), with 4500 mg/L glucose, L-glutamine, and sodium bicarbonate, without sodium pyruvate, liquid, sterile-filtered, 1% Antibiotics—penicillin/streptomycin (Sigma) made up of 10,000 units’ penicillin and 10 mg streptomycin/mL, sterile-filtered, Bio-Reagent and 10% Fetal Bovine Serum (Sigma) were used to prepare the complete culture medium (CCM).

#### 3.1.3. Consumables

Most of the non-chemical consumables as listed below were purchased from Fisher Scientific (Pittsburgh, PA, USA) and the electrodes for impedance measurements were purchased from Applied Biophysics Inc. (New York, NY, USA). 

##### Consumables for Biological Sample Preparation and Test

Biological Sample Preparation requires precision and careful handling. This entails use of different materials such as Sarstedt Serological pipettes (Sarstedt AG & Co. KG, Nümbrecht, Germany), Culture dishes TC-Schale (Standard Sarstedt AG & Co. KG), 12 and 6 well Culture Plates (Sarstedt AG & Co. KG), Conical centrifuge tubes (Thermo Scientific, Waltham, MA, USA), Petri dish (Sarstedt AG & Co. KG), Universal Fit pipette tips and microtubes (Sarstedt, Corning Inc., Newton, NC, USA) Cell counter 40 μm sensor (Scepter™ 2.0, Millipore Sigma, Burlington, MA, USA).

##### Consumable for Impedance Analysis

Recording and analysis of impedance were made possible by using two different electrode arrays. The Electrode Array (type 1) ECIS, PC (Clear polycarbonate substrate) 1E, Diameter of electrode (central hole), 250 μm and Electrode Array (type 2) ECIS, PCB (Non transparent Printed Circuit Board) IE Diameter of electrode (central hole), 250 μm of 0.049 mm^2^ were purchased at Applied Biophysics Inc, NY, USA.

#### 3.1.4. Equipment

All equipment used in this project for biological sample preparation and analysis, impedance measurement and analysis and microscopic analysis are listed below. 

##### Required Equipment for Biological Sample Preparation and Test

The N2a cells were incubated in a Heracell 150i incubator (Thermo Fisher Scientific). Chemicals were prepared and maintained in Forma refrigerator (Thermo Scientific) and stored at Forma 900 series freezer. All cell culture techniques, including preparation and aliquoting, of the solutions, were performed in a laminar flow unit 1300 series A2 (Thermo Scientific). The HiFlow, F19917-0250 Vacuum Aspirator Collection System (SP Scienceware), Isotemp Digital 2320 Water Bath (Fisher Scientific), and Sorvall ST 8 Lab Centrifuge (Thermo Scientific) were also used. The Fisherbrand™ Analog Vortex Mixer (Fisher Scientific) was used during the preparation cell-dye mixture of TBDE Assay. Equally important equipment used during the preparation was Quintix® Analytical Balance (Sartorius), Hanna Checker® pH meter (Sigma Aldrich). Sceptre™ 2.0 Handheld Cell Counter (Millipore Sigma) was used during the cell counting.

##### Required Equipment and Accessories for Microscopic Analysis

Images were captured with the used of Fisherbrand™ Inverted and phase contract Microscope (Fishers Scientific) with an attached Education™ Motic D-Moticam 1080 Digital HDMI camera (Fishers Scientific). BLAUBRAND® Neubauer Hemocytometer (Millipore Sigma) was used during the manual counting and visualization of live and dead cells.

##### Required Equipment for Impedance Analysis

Autolab PGSTAT101, FRA32M electrochemical impedance spectroscopy (EIS) module (Metrohm, Herichaut, Switzerland) was the primary equipment for the impedance analysis.

#### 3.1.5. Software

The following software was utilized in obtaining data and for analysis purposes. The Scepter™ 2.0 Software Pro User Interface (Millipore Sigma) was used for cell concentration and volume determination. Concurrently, Motic 2.0 software (Fishers Scientific) was used to record the images. NOVA 2.0 software (Metrohm) was used to record and analyze impedance. Excel (Microsoft) had been used for displaying and analyzing the data. 

### 3.2. Methods

**Figure 2:**
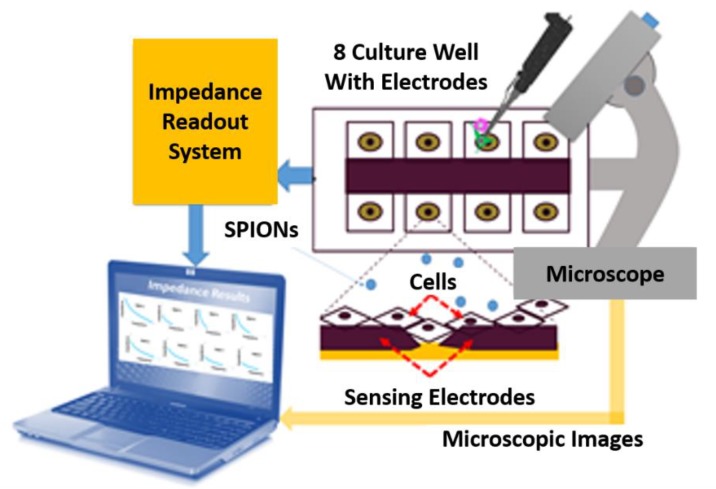
Scheme of proposed experimental setup including an array of 8 sensors incorporated with cell culture wells. These electrodes are connected to a computer through an impedance readout system. The cells are loaded by a standard pipette and observed under a microscope.

In this work, the cells were cultured with different concentration of SPIONs (0, 25, 50, 100, 200 and 300 µg/mL) in the traditional Petri dish and in the ECIS electrode array as seen in Figure 2. The cell viability, cell morphology analysis, and the impedance-based cell–surface attachment in the presence of SPIONs are measured using various methods. The details of the measurement results are shown in the next section.

#### 3.2.1. Sample Preparation and Biological Test

In this subsection, the protocols related to the preparation of samples including biological cells, SPIONs and their related biological assays were put forward. 

##### Preparation of SPIONs with Different Concentration

After thorough calculations, the 300 µg/mL, 200 µg/mL, 100 µg/mL, 50 µg/mL, and 25 µg/mL concentrations of SPIONs were prepared by weighing 6, 4, 2, 1, 0.5 mg of SPIONs respectively using the Sartorius Quintix^®^ and dissolved in the cell culture medium (CCM) to reach a total volume of 20 mL of the mixture. Then, SPION solutions were transferred into a conical tube containing a small amount of the CCM. A vortex mixer with a dimension of 20.3 × 14 × 12.2 cm was set at the speed knob 9 with a speed of 3200 rcf to disperse and dissolve the SPIONs for 15 minutes. After that, CCM was added to reach the desired volume used for the test. 

##### Cell Culture and Maintenance

N2a cells were grown in complete culture medium (CCM) containing Dulbecco’s Modified Eagle’s Media (DMEM) supplemented with 10% Fetal Bovine Serum (FBS) and 1% antibiotic solution Penicillin-Streptomycin (PS). The cells were maintained in a Heracell CO_2_ incubator with 5% CO_2_/37 °C temperature. 

As a part of maintenance, the cells were passaged twice a week. Once 90–100% confluency was reached, the cells were washed twice with 5 mL pre-warmed PBS, treated with 1 mL pre-warmed trypsin-EDTA and incubated for 1–5 min for the cells to detach from the substrate. To ensure the detachment, the cells were viewed under the microscope. 1 mL of CCM was added and transferred to a 15 mL conical centrifuge tube containing 1 mL of CCM. The mixture was put in the centrifuge with a speed of 2500 rpm in 2 min. Afterwards, the supernatant was removed, and the cells were resuspended with pre-warmed CCM in a culture Petri dish. 

##### Cell Concentration Preparation and Inoculation

After counting the cells using hemocytometer, the concentration of 2.5 × 10^5^ cells/mL was prepared by diluting N2a cells with CCM and SPIONs mixture. The cells were seeded in both a non-transparent (PCB model) and transparent (PC model) 8-well ECIS array. It is noteworthy that a single concentration of cell was prepared and used for all the tests.

##### Preparation for TBDE Test Mixture

Trypan Blue Dye Exclusion (TBDE) was used to determine the number of alive and dead cells after 72 h of exposure into the different concentrations of SPIONs. When the cell is viable, its membrane does not allow penetration of the dye leaving the cells to appear rounded, clear and shiny under the microscope, which distinguishes it from a dead cell that enables penetration of the blue dye. A 100 µL of the cell samples were diluted and gently mixed with an equal volume of trypan blue in a microtube, and it was set aside in room temperature for 3–5 min. 

##### Cell Counting and Cell Viability Test

After 72 h cell culture, the cells were collected using the standard trypsinization method. A randomized, double-blind method was carried out for the preparation of the dilution of the trypan blue dye and cell suspension to avoid bias. Each Eppendorf tube was labelled and covered with tape. Three biological replicates were prepared for every concentration. BLAUBRAND^®^ Neubauer Hemocytometer (Millipore Sigma) was used to count the number of dead and live cells. The coverslip was slightly moistened with ultrapure water and slid it into the hemocytometer, gently avoiding the formation of bubbles. The mixture of cells was loaded by 10 µL under the coverslip. The hemocytometer was placed under the inverted microscope using a 10× objective lens. The number of live (unstained cells) and dead (stained) cells were counted in all the five areas with 16 squares.

The Scepter™ 2.0 Handheld Cell Counter (Millipore Sigma) was also used to measure the concentration of cells. A cell suspension diluted with PBS reached a total volume of 100 µL. The mixture was put in a 2.0 mL microcentrifuge tube. When using the Scepter cell counter, a 40 µm sensor was attached and submerged into the mixture. After a 50 μL sample was drawn into the channels of the sensor, the cell concentration was displayed on the screen, and the files were transferred into the computer for analysis.

#### 3.2.2. Microscopic Methods

An inverted microscope equipped with motic 2.0 camera and various objectives (e.g., 4×, 10× and 20×) was used to capture microscopic images in eight different times. These microscopic images were used to monitor cellular morphological changes and likely their adhesion, growth, and differentiation, in the presence of different concentrations of SPIONs.

#### 3.2.3. Electrical Methods

##### The Principle of Impedance Spectroscopy Technique for Cellular Analysis

**Figure 3:**
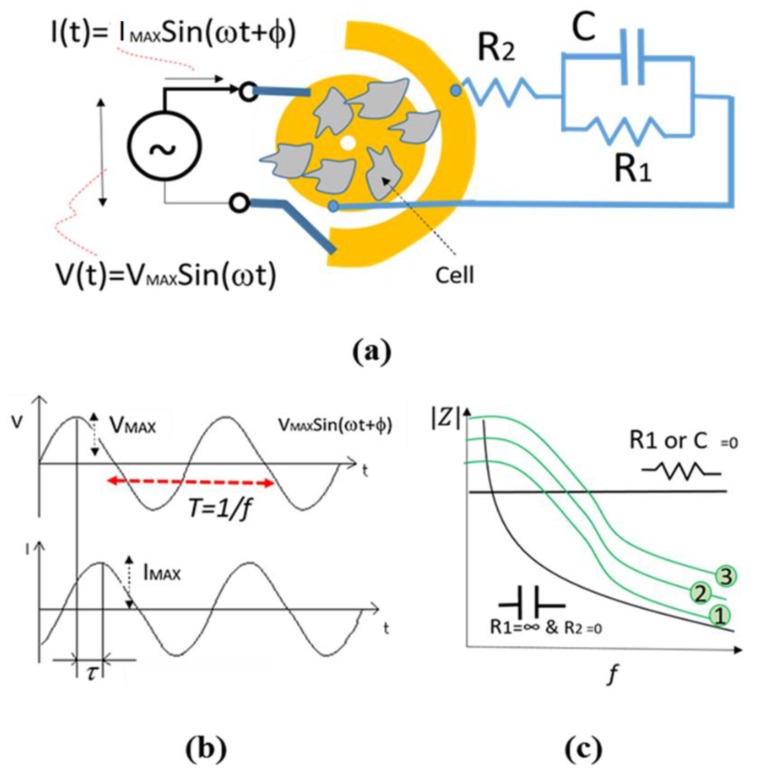
Principle of impedance measurement: (**a**) schematic of electrode and its equivalent circuit, (**b**) the sine wave voltage and current signals and (**c**) the impedance frequency response of electrode.

Impedance spectroscopy is a technique that measures the electrical impedance between two close electrodes exposed to the chemical or biological materials. Impedance is a combination of resistive and capacitive properties of the material. The equivalent circuit of the electrode exposed to the cells in culture can be represented with the schematic shown in Figure 3. 

The magnitude of impedance between the electrodes can be represented by Equation (1).

(1) $$\left|Z\right|=\sqrt{\frac{{\left(R_{1}+R_{2}\right)}^{2}+{\left({\omega C}_{1}R_{2}R_{1}\right)}^{2}}{1+{\left({\omega C}_{1}R_{1}\right)}^{2}}}$$

where R_1_ and C represent the resistance and capacitance properties of cells attached to the electrodes, respectively. Also, R_2_ represents the resistance of connectors as well as medium. *f* is the frequency of sine shape electrical voltage applied to the sample and resulted in an electrical current with the same frequency (see Figure 3b). *f* is equal to the inverse of the time T. Indeed; the impedance is equal to the magnitude of V_MAX_/I_MAX_ where both V_MAX_ and I_MAX_ are the amplitude of electrical voltage and current signals as seen in Figure 3. As seen in Figure 3c, depending on the type of medium and biological material, the equivalent circuit can be a simple resistor or capacitor. However, the equivalent impedance magnitude is very similar to the green curves shown in Figure 3c so that by increasing the impedance, the curve 1 moves to curve 2 and then 3. 

In other words, the attachment and confluence of cells on electrodes and in between the electrodes result in higher impedance. It is noteworthy that φ and τ are the time and phase differences respectively, as seen in Figure 3b so that φ = 2πτ/T = 2π*f*τ. In this project, we only use the magnitude of impedance. Therefore, the phase differences are not taken in our calculations. 

##### Impedance-Based Cellular Analysis

**Figure 4:**
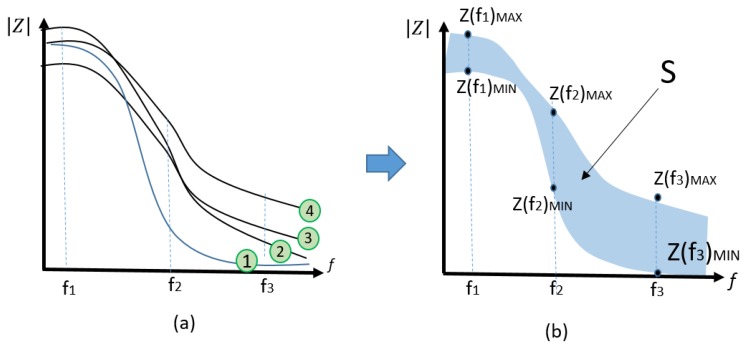
Illustration of (**a**) multi-curves impedance spectroscopy results and (**b**) the covered surface area S.

The cell attachment and growth above the electrodes can be monitored by measuring the impedance in between the electrodes [85,86,87,88,89,90,91,92,93,94,95,96,97,98,99,100,101,102,103]. The attachment of cells above electrodes can increase the dielectric properties and decrease the conductivity; therefore, the amount of impedance in all frequencies is increased as seen in Figure 4a. This figure shows the increase of impedance of electrodes underneath of cells in culture over time. 

##### Maximum Surface Area

As the first impedance analysis method, we use the maximum variation of impedance as a healthy factor of cells in the presence of SPIONs. As seen in Figure 4b, the surface area S represents the maximum change and is calculated by Equation (2)

(2) $$S=\overset{f_{\mathrm{MAX}}}{\underset{f_{\mathrm{MIN}}}{\sum }}\frac{\left(Z{\left(f\right)}_{\mathrm{MAX}}-Z{\left(f\right)}_{\mathrm{MIN}}\right)\cdot \Delta f}{f_{\mathrm{MAX}}-f_{\mathrm{MIN}}}$$

By knowing ∆f is the minimum frequency change and f_MAX_–f_MIN_ refers to the range of scanned frequencies, therefore (f_MAX_–f_MIN_)/∆f is equal to the number (N) of the different frequencies where the impedance is measured. In other words, S can be obtained from the following equation.

(3) $$S=\overset{N}{\underset{1}{\sum }}\frac{\left(Z{\left(f\right)}_{\mathrm{MAX}}-Z{\left(f\right)}_{\mathrm{MIN}}\right)}{N}$$

As an example, Table 3 shows the parametric impedances measured in different frequencies and times. It is assumed that the cells are mixed with an arbitrary concentration of SPIONs. The maximum and minimum values of impedances are obtained and used to calculate the impedance change ∆Z = Z(f)_MAX_−Z(f)_MIN_ in different times and frequencies as shown in the table. As a result, a column of various ∆Z is obtained. Based on Equation (3), the average of all numbers in this column is equal to S, and consequently, it shows the maximum variation of impedance. 

As previously mentioned, S is equal to the average of Z_MAX_−Z_MIN_ in various frequencies. In the next section, we also obtained the variance and standard deviation of Z_MAX_−Z_MIN._ Additionally, one may argue the normalized values of S can be related to the concentration of SPIONs. To show this, we also calculated the average or standard deviation of all impedances measured in each frequency (f_1_–f_N_) as seen in Table 4 that is the continuation of Table 5. In this situation, instead of Z_MAX_−Z_MIN_(f), Z_MAX_−Z_MIN_(f) /AVG(f) and STD(f) should be calculated and shown in a column. Finally, the average, variance, and STD of this column can be calculated to obtain a kind of normalized S. It is noteworthy that when using these calculation methods, we try to quantify the effect of SPIONs on cells in culture. 

##### Electrical Model

As per Equation (1) and as seen in Figure 3, an equivalent circuit with specific values of R_1_, C and R_2_ can be fitted with the impedances measured in each time (T1–T8) in a range of frequency, in a specific value of SPIONs. In this method, a software called NOVA 2.0 is used to find the optimum values of an equivalent circuit for each range of frequencies. In this method, various values of C, R_1,_ and R_2_ for various concentrations of SPIONs were obtained at different times to study the effect of SPIONs on the cell culture over time.

##### Impedance Measurement Assay

Using Metrohm Autolab, the impedance spectroscopy of the eight wells were measured over different times (0, 2, 4, 6, 8, 24, 48 and 72 h). The impedance was monitored using a frequency ranging from 0.1 Hertz (Hz) to 100,000Hz with alternating current (AC) 100 mV voltage. The electrode array was interconnected into the impedance system using copper wires. The impedance measurement results were saved into Excel files for further analysis and display. 

Before loading the ECIS array with cells, it was cleaned with PBS, rinsed with ultrapure water and electrodes were pre-conditioned by flooding each well with 200 µL of cysteine solution for 10 min and equilibrate with DMEM. In some circumstances, the electrodes were also further cleaned and treated in an oxygen plasma for 60 s.

After calibration, a monodisperse 2.5 × 10^5^ cells/ml concentration of cell was inoculated separately in each of the six wells. During the inoculation, the cell suspensions were agitated to prevent settling of cells from the bottom of the tube. Meanwhile, the remaining wells were filled with the CCM and different concentrations of SPIONs without cells. After each test, the ECIS device was put back in the incubator. 

In this section, the details of materials and methods were elaborated. The main methods, including biological, microscopic and impedance methods, were used to study the effect of SPIONs on the cells. These methods applied to a large number of samples as demonstrated and discussed in the next section. This study takes us a step closer to assessing the need for high throughput cellular analysis for various applications for toxicity studies. 

## 4. Results and Discussions

In this section, the experimental results related to biological, microscopic and impedance methods are separately demonstrated and discussed in Section 4.1, Section 4.2 and Section 4.3 as summarized below.

- Biological method: The cell viability tests were performed using the trypan blue exclusion assay. This technique was used to count the number of viable cells after 72 h (T8) of exposure.
- Morphological method: The microscopic images of the N2a cells were captured to compare the treated and untreated cells. The treated cells were the cells mixed with SPIONs with different concentrations (C2–C6). The N2a cells were cultured in an incubator.
- Electrical method: The attachment of cells and SPIONs above electrodes can change the impedance as described in Section 3. The impedance spectroscopy of cells in control (C1) and with the presence of SPIONs (C2–C6) are measured in different times (T1–T8) by hypothesizing that the effect of SPIONs on cells can be tracked using the recorded impedances.

Two series of cell culture experiments were performed using PC and PCB electrode arrays, as mentioned in Section 3. Each set of experiments includes three different trials (TR1, TR2, and TR3). In each trial, experiments were replicated three times (G1–G3). In each group (G1, G2 and G3), six different concentrations (C1–C6) of SPIONs were mixed with cells and cultured in the incubator for 72 h. The initial cell concentration, which is 2.5 × 10^5^ cell/mL the cell viability (V), was measured after 72 h. The microscopic images (M) and impedance measurements (I) were obtained in eight different times (T1–T8). All experiments were repeated without cells to control the results. The entire experiment was performed for six months.

Meanwhile, the number of experiments per chamber is TR·CC·T·C·G=864. The number of experiments using PC and PCB electrode can reach 1728. By knowing that the microscopic images of experiments related to PCB electrodes were performed on a Petri dish, the total number of the experiment should be added using approximately TR × G × C = 54 Petri dishes. 

### 4.1. Biological Effects

**Figure 5:**
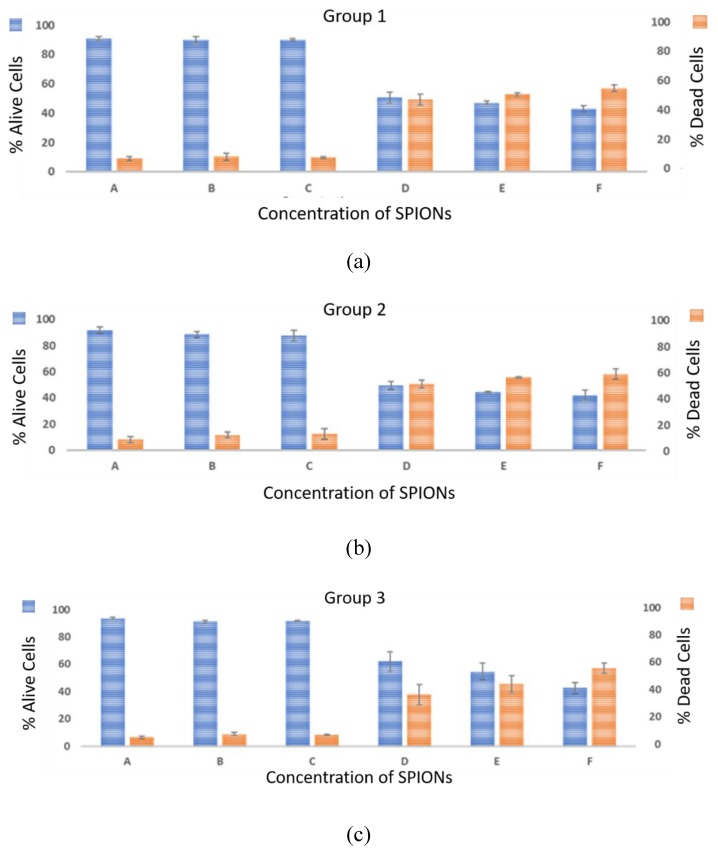
Viability Results. The percentages of both viable and dead N2a cells in the three different (**a**) group 1, (**b**) group 2 and (**c**) group 3 were graphed concerning six different categories A–F. 0, 25, 50, 100, 200 and 300 µg/mL SPIONs concentrations in the first trial.

This section demonstrates the effect of SPIONs on the viability of cells. Figure 5 shows the percentages of alive and dead cells in the presence of six different concentrations of SPIONs (0, 25, 50, 100, 200 and 300 µg/mL) in the cell culture. 

In each group, the mean value of the three replicates was calculated for both alive and dead cells. Consistent with the results shown above, an inverse relationship between the concentration of SPIONs and cell viability was found. As seen in Figure 5, for C ≥ 50 µg/mL, the cell viability is high and almost invariant. On the other hand, the cells exposed to C ≥ 300 µg/mL SPIONs show the highest percentages of cell mortality. The same trend is observed using the PC array device. Low cell viability with percentages of 59, 47, 40% in higher concentrations of SPIONs, 100 µg/mL, 200 µg/mL, 300 µg/mL respectively is observed in PCB array device. All three trials on PCB and PC had shown that by increasing the concentrations of SPIONs, the cell viability was decreased, which may be due to the increased toxicity effect of SPIONs on cells. These results are in agreement with the results observed by Naqvi et al. [102,104] toxicity is amplified by higher doses of NPs.

Also, from the 25000 initial cell concentration at T1, all N2a cells in control with no SPIONS (C1) and those that treated with different concentrations of SPIONs (C2–C6) manifested an increased cell concentration after 72 h (T8). However, C4, C5, C6 showed lower cell concentration growth compared to C1. Relative to C1, the percent differences of cultured cells in C4, C5, C6, were 59%, 44% and 53% respectively, while 24% and 16% difference was observed at C2 and C3, respectively. Higher concentrations of SPIONs showed higher differences relative to the control, as shown in reference [105].

**Figure 6:**
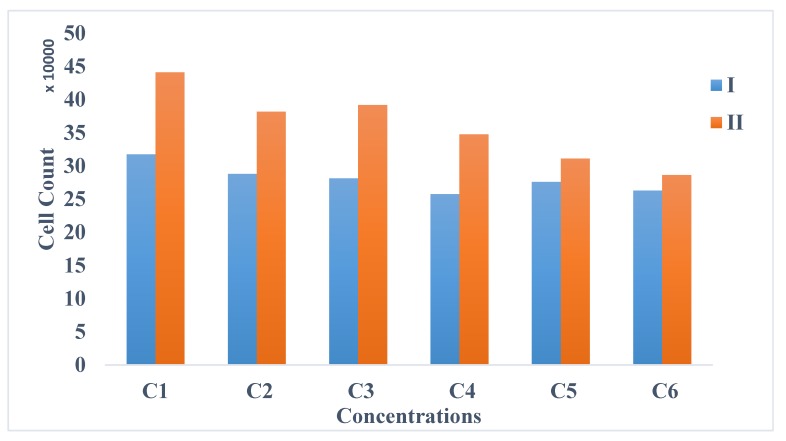
Comparison of TBDE and the cell counter. The trypan blue dye exclusion (I) showing a lower number of cells counted in all the concentrations of SPIONs (C1 = 0, C2 = 25 µg/mL, C3 = 50 µg/mL, C4 = 100 µg/mL, C5 = 200 µg/mL, C6 = 300 µg/mL) compared to the Scepter Handheld Cell counter (II).

Although the viability test was mainly determined using the Trypan Blue Dye Exclusion (TBDE), the number of cells were also counted using a Scepter™ 2.0 Handheld Cell Counter (see [105]). Figure 6 shows the mean values of counted cells using the TBDE and Scepter counter. Additionally, this figure shows a higher number of cells measured by Scepter cell counter compared to the one obtained in TBDE. The difference might be due to the presence of SPIONs’ aggregates along with the cells being detected, considering that Figure 6 shows the concentration for the total event, not the gated concentrations. Moreover, the calculated percent coefficient of variation (% CV) of the cell concentration in all concentrations (C1–C6) using TBDE is 8.85, 6.05, 8.69, 4.75, 6.32, 10.80, respectively. Relative to TBDE, using the resceptre cell counter, the percentages of CV in C1–C6 are 40.48%, 27.13%, 20.04%, 30.65%, 40.38%, and 56.88%, respectively. Comparing % CV results obtained using two different techniques, TBDE results shows less variation and consequently high accuracy, relative to the resceptre cell counter’s results. 

### 4.2. Morphological Effects

**Figure 7:**
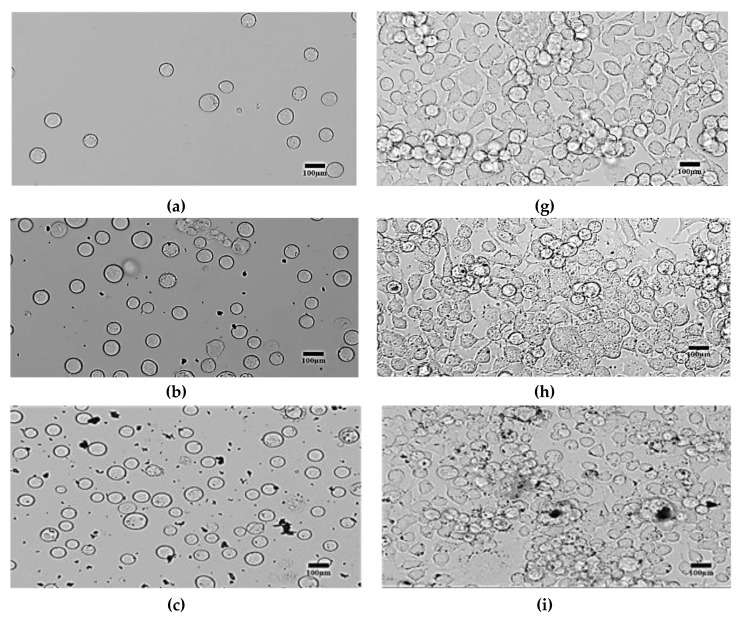

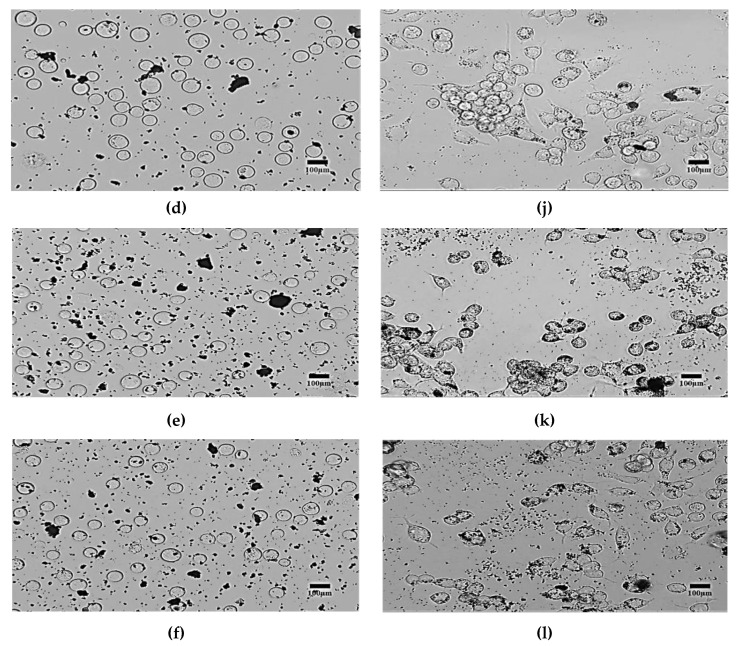
Photomicrographs of untreated and treated N2a cells at the initial time (T1) and after 72 h’ (T8) incubation. Images in **a**, **b**, **c**, **d**, **e**, and **f** were taken at T1 while **g**, **h**, **i**, **j**, **k**, **l** at T8. a, g = untreated N2a; b, h = treated with 25 µg/mL; c, i = 50 µg/mL; d, j = 100 µg/mL; e, k = 100 µg/mL; f, l = 100 µg/mL SPIONs concentrations. Scale bar 100 µm.

In this section, the adhesion, confluence and morphological changes of cells are evaluated using microscopic images. A motion 2.0 camera captured the microscopic changes of cells cultured on transparent electrodes and Petri dishes at different times (T1–T8). The images were captured from the same location of electrodes or Petri dishes and the same magnification (20 × 10 = 200). Figure 7a–f shows the N2a cells treated with C1-C6 concentrations of SPIONs respectively at T1 while Figure 7g–l shows the same cells incubated with the same concentrations of SPIONs at T8. These microscopic images are used to observe the growth, proliferation, and formation of neurite extensions of the N2a in the presence of various concentrations of SPIONs. In general, based on the results shown in Figure 7, the growth and proliferation of N2a are faster in the absence of SPIONS. A similar observation was pointed out in the study presented by Eustaquio and Leary (2012), where proliferation and differentiation of cells are affected by their exposure to nanoparticles [11]. Similarly, the decreased proliferation, brought about by an increasing amount of SPIONs, was also observed in the study performed by Lindemann et al. [106]. Also, Chen et al. (1997) pointed out that modifying the environment of cells such as cell substrate including the medium may alter cell behavior and shape, which can lead to decreased adhesion and increased cell death [107].

In this experiment, each Petri dish and PCB/PC culture array were seeded with the same concentration of cells. As shown in Figure 7a–f, the cells had a round shaped at T1. At T8, the cells were completely adhered, differentiated and somehow developed neurites as seen in Figure 7g–l. As manifested in Figure 7g–h, the surfaces of Petri dishes were completely covered with cells where the concentrations of SPIONS were C1 and C2, respectively. This shows the highest cell confluence in the Petri dishes. However, Figure 7j–l shows less cell confluence on the surfaces of Petri dishes. Thus, it seems that longer neurites were generated to connect the nearby cells.

Additionally, by increasing the SPIONs, the size of SPIONs clusters is also increased as seen in Figure 7j–l.

**Figure 8:**
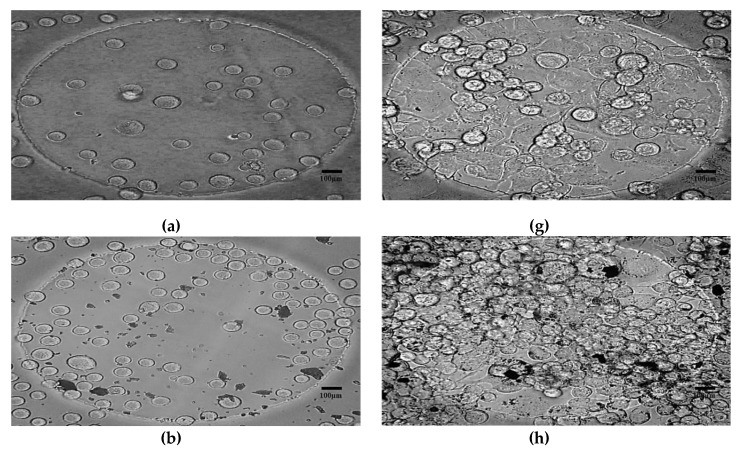

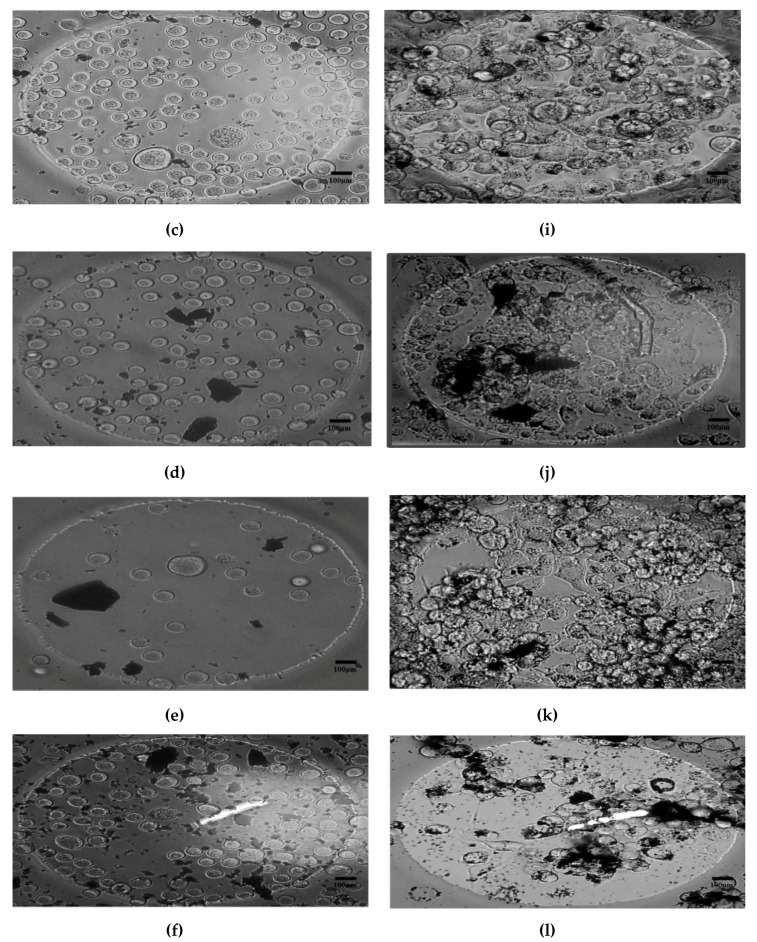
Photomicrograph of N2a cells exposed to the different concentrations of SPIONs on the surface of a 250μm diameter electrode on a clear polycarbonate substrate. Images in **a**, **b**, **c**, **d**, **e**, and **f** were taken at T1 while **g**, **h**, **i**, **j**, **k**, **l** at T8. a, g = untreated N2a; b, h = treated with 25 µg/mL; c, i = 50 µg/mL; d, j = 100 µg/mL; e, k = 100 µg/mL; f, l = 100 µg/mL SPIONs concentrations. Scale bar 100 µm.

Similarly, Figure 8a–l shows the growth of N2a cells on the surface of the electrode at T1 and T8 in different concentrations of SPIONs. Figure 8g and h show 95 to 100% cell confluence after 72 h of cell culture in an incubator. It is noteworthy that the optically transparent electrode array or so-called PC electrode array allowed us to count the number of cells using an optical microscope and measure the electrical impedance. The morphological changes as a result of the interaction of N2a to the different of SPIONs from T1–T8 are shown in reference [105].

### 4.3. Impedance Effects

As previously mentioned, in this paper, 1728 experiments were performed using PC and PCB electrodes. In each experiment, eight impedance measurements were performed at T1 to T8. Therefore, the number of the recorded complex impedance Z_R_ + jZ_I_ values in 60 different frequencies is about 103,680, where ZR, Z_I_ are real and imaginary values of impedance in each frequency, as seen in Table 6. In this table, the magnitude of impedances ($\sqrt{Z_{R}^{2}+Z_{I}^{2}}$) is shown at different times (T1–T8). In this section, the results are demonstrated and discussed in three different forms—impedance spectroscopy, time-averaged impedance spectroscopy, and integration methods, as described in Section 3.

#### 4.3.1. Impedance Spectroscopy

This subsection includes the direct measurement of impedance spectroscopy at different times and different concentrations of SPIONs. Table 6 partially shows the magnitude of impedances at T1–T8 in various frequencies ranging from 0.1 Hz to 100,000 Hz. 

**Figure 9:**
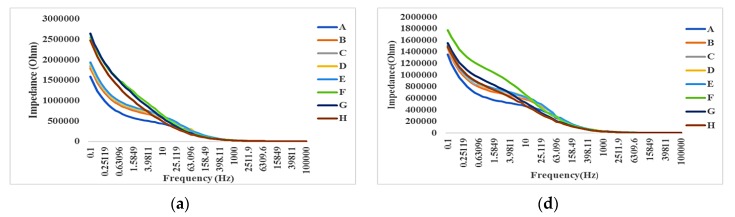

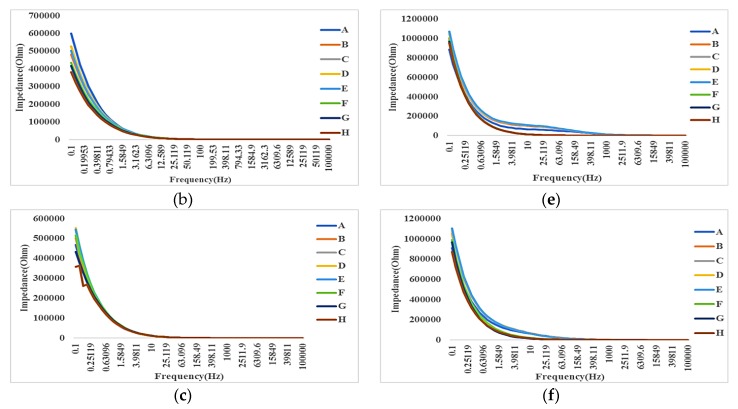
Impedance spectroscopy at different times T1 = A, T2 = B, T3 = C, T4 = D, T5 = E, T6 = F, T7 = G and T8 = H at different SPIONS concentrations: (**a**) C1, (**b**)C2, (**c**) C3, (**d**) C4, (**e**) C5 and (**f**) C6.

Figure 9a shows the impedance spectroscopy results at different times (T1–T8) using the same concentration of SPIONs (C1). Similarly, Figure 9b–f shows the impedance spectroscopy results at C2–C6, respectively.

#### 4.3.2. Time-Averaged Impedance Spectroscopy

**Figure 10:**
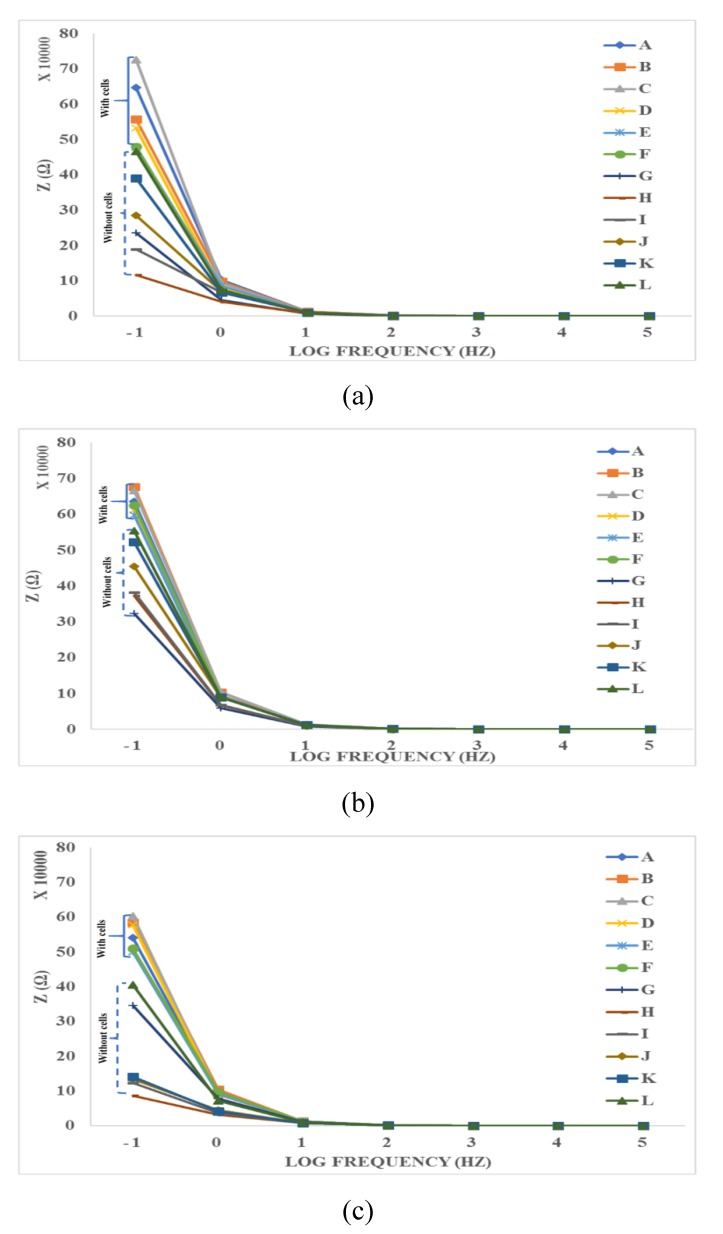
Impedance Spectroscopy Readings of the control and different concentrations of N2a-SPIONs using a range of frequency from the log −1 to 5 (0.1 to 100000Hz). A 2.5 × 10^5^ cells/mL concentration was used for the three groups((**a**) group 1, (**b**) group 2 and (**c**) group 3). Legend shows the different concentrations of SPIONs and the control groups where A = N2a (control), B = 25 µg/mL with cells, C = 50 µg/mL with cells, D = 100 µg/mL with cells, E = 200 µg/mL cells, F = 300 µg/mL with cells, G = CCM (Control), and H = 25 µg/mL without cells, I = 50 µg/mL without cells, J = 100 µg/mL without cells, K = 200 µg/mL without cells and L = 300 µg/mL without cells.

Figure 10 shows the time-average of impedance spectroscopy in each frequency where A = N2a (control, C1), B = C2 with cells, C = C3 with cells, D = C4 with cells, E = C5 with cells, F = C6 with cells, G = CCM (Control, C1, without cell), and H = C2 without cells, I = C3 without cells, J = C4 without cells, K = C5 without cells and L = C6 without cells. Each curve in the groups 1, 2 or 3 (e.g., orange color, B) display the mean value of 8 impedance curves related to the same samples, in 8 different times. Also, Figure 10 shows the time-average effect of various samples on the impedance. The measurements were categorized into three different groups 1–3 (from the beginning to seventy-two hours of incubation). The highest impedance was manifested by the positive control group (N2a, with cells) while the lowest impedance value was observed from the negative control group (CCM, without cells). Based on the results shown in all groups in Figure 10, the presence of cells with or without SPIONs increase the impedance. This might be due to adhesion between the cell and electrodes. In the other hand, the lower the SPIONs concentration, the lower the impedance is expected to be in the sensing electrodes without the cells. This might be because the lower the SPIONs concentration becomes, the lower dielectric property can be expected to be. Another interesting outcome in the curves shown in Figure 10a is that the maximum impedance change is about 0.48–0.75 MΩ for SPIONs with concentrations ranging from 0 to 300 µg/mL. Therefore, the resolution of this measurement is about a 0.9 kΩ impedance change due to a 1 µg/mL SPIONs change. Based on the results shown in Figure 10b,c, the resolutions can be calculated similarly. The mean value of resolutions calculated in all three groups is about 520 Ω mL/µg. The highest impedance is clearly manifested among the lowest concentrations of SPIONs, C B and A mixed and cultured with cells. Meanwhile, the lowest impedance values were observed in the negative control, CCM (G) and CCM-SPIONs mixtures without cells (H-L). 

#### 4.3.3. Integration Methods

**Figure 11:**
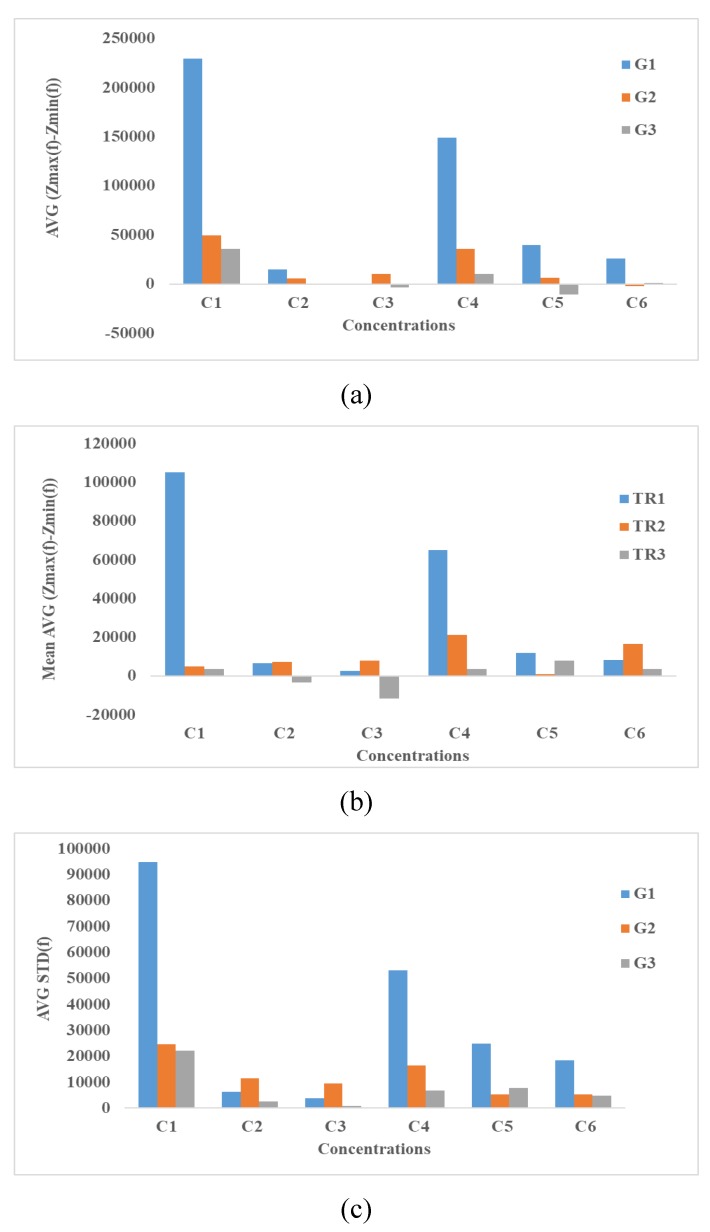
Integrated Impedance Spectroscopy results using equations: (**a**), (**b**) and (**c**) at different concentration of SPIONs (C1–C6) and three different groups (G1–G3).

As described in Section 3, the variation of surface area (∆S) under the impedance spectroscopy curves can be used as a measure to study the effect of cultured cells in the presence and absence of SPIONs. Figure 11a shows the calculated ∆S from the impedance spectroscopy results at six different SPIONs concentrations (C1–C6) at three different repeats (G1–G3), where the equation Z_MAX_−Z_MIN_ was used. Similarly, Figure 11b,c show the calculated ∆S using the equations Z_MAX_−Z_MIN_(f) of TR1, TR2, TR3 and STD (Z_0_(f_1_) … Z_72_(f_N_), respectively.

Based on the results shown in Figure 11, the higher the concentration, the lower ∆S is observed where the concentrations of SPIONs are C1, C2 or C3. Figure 11a–c shows a significant increase of ∆S at C4. Also, by increasing the SPIONs concentration (C5, C6), ∆S is decreased. Interestingly the same spike at C4 is observed in Trials 2 and 3. It is noteworthy, even though three different equations are used in Figure 11a–c, that the spike at C4 can be observed. One may argue that the shape and dimensions of electrodes, the material, size and concentration of nanoparticles can be considered as the main factors in the electrical models that have resulted in the creation of a spike at C4. Indeed, there are many other factors, such as the culture medium and even the alive cells that can affect the results shown in Figure 11a–c. A general justification can be provided using Figure 7j–l. As seen in Figure 7j, the presence of SPIONs with a high concentration (C4) has significantly resulted in decreasing the cell confluence. Instead of cells, the surface of the electrodes was coated with SPIONs. Therefore, it is expected that the charged SPIONs bond with the surface of electrodes and significantly increase the double layer capacitance and consequently increase the impedance in low frequencies. This change of impedance can justify the spike at C4. In the other hand, assuming that the SPIONs fully cover the surface of electrodes at C4, the increase of SPIONs concentration may result in creating larger aggregates and in affecting the cell attachment or cell growth as seen in Figure 7k,l. 

#### 4.3.4. Equivalent Electrical Circuit’s Method

**Figure 12:**
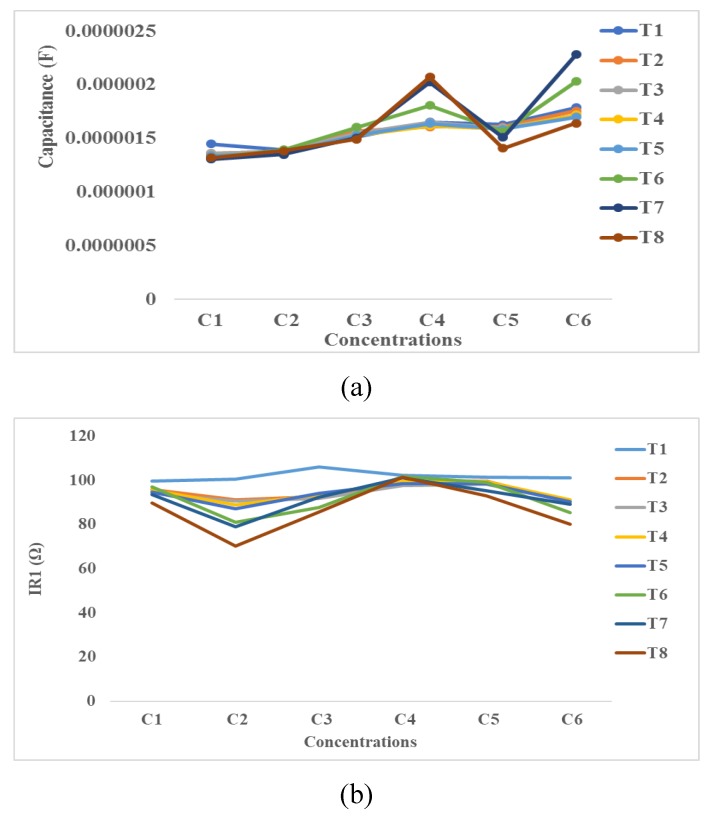

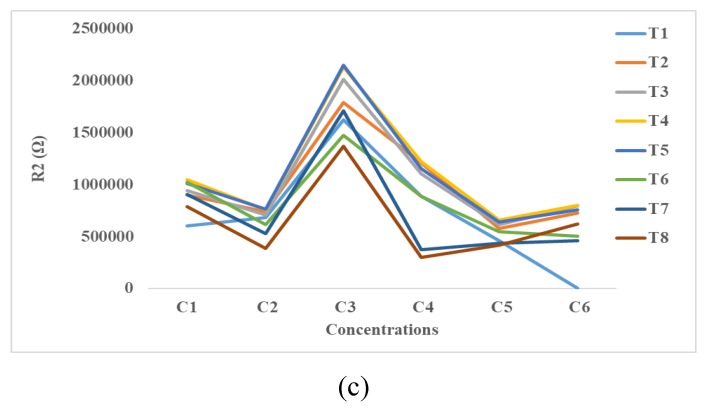
Equivalent electrical circuit including: (**a**) capacitance, (**b**) series resistance and (**c**) parallel resistance/impedance.

Figure 12 shows the variation of capacitance, the series, and parallel resistances as a function of SPIONs concentration at T1, T2, T3, T4, T5, T6, T7, and T8. As seen in these Figures, the capacitance (C), series resistance (R1), and parallel resistance (R2) are in the range of 0.5–2.5 µF, 65–110 Ω, and 25–225 KΩ, respectively. Based on these results, the variation of SPIONs concentration or time do not significantly affect R1 and C. This is because R1 is proportional to the resistance of bulky medium that is highly conductive and the variation of SPIONs concentrations does not significantly change this conductivity. In the other hand, C is proportional to the double layer capacitance (DLC). DLC is affected by the attachment of cells or the distribution of SPIONs on the surface of electrodes. DLC is almost constant because of the higher attachment of cells, the lower surface area coated by SPIONs and vice versa. R2 in parallel with C changes over the times T1–T8. Similarly, R2 changes over the concentration of SPIONs (C1–C6). This might be due to the attachment or the deposit of molecules in the culture medium above the electrodes. 

## 5. Future Discussion

Validating toxicity results depends on the sample size and replications. However, generating data with a large number of replicates is one of the challenges that any researcher needs to address. This section reveals the difficulty in obtaining a high volume of data in terms of economical and time elements involved in the study. This section also provides a glimpse of the ideal characteristics of the high throughput device for the future. 

### 5.1. Economical Assessment

This subsection provides an estimated cost of the materials and chemicals used in the study and any high throughput toxicity assay. The estimated cost for performing 1728 experiments is about $2300. In a clinically relevant cytotoxicity study, by assuming C = 24 different concentrations of SPIONs with more than G = 12 times replicates and CC = 5 different cell concentrations, the number of experiments will approximately be equal to $184,000. The figures would prove a financial challenge in performing a number of experiments that obtain sufficient data to validate results and conclusions about toxicity studies. The high throughput platform containing a large number of micro-scale chambers enables parallel analysis that significantly decreases cost and the required time, as described in the next subsection.

### 5.2. Time Assessment

The experimental portion of this study involves different trials and replications that are time-consuming. 

In this paper, in addition to biological and microscopic methods, a label-free impedance spectroscopy method was used as s new alternative technique for cellular analysis. The impedance readouts were recorded from eight different times (T1–T8) for each one six different (C1–C6) SPIONs concentrations with and without cells. As previously mentioned in chapter 3, this generates 1728 curves in almost 60 frequencies. In other words, this approximately counts up 100,000 impedance magnitude numbers. By assuming each number takes 5 s, the experiments can be completed after 6-days of continuous work. The required time for a clinically relevant cytotoxicity study is 80 × 6 = 480 days. In other words, it takes more than 16 months to complete the experiments. A high throughput platform containing at least 100 chambers in parallel for cell culture and impedance analysis can decrease the required time to less than 480/100 ~ 5 days.

### 5.3. High Throughput Analysis Device for the Future

Based on a large number of data generated to establish the interaction of SPIONs to the N2a cells, we anticipate that an automated high throughput screening system will be developed with highly sensitive electrodes to capture more complex activities of the cells. The high throughput analysis device can generate data faster. Since this study focused on the effect of SPIONs for a single type and concentration of cell, this can be further repeated using other types and concentrations of cells. Images depicting the morphology of the cells should be further examined to see potential pathologically significant results.

## 6. Conclusions

In this section, we demonstrated and discussed the viability test results using TBDE and Scepter cell counter. Based on the viability test results, there are higher chances for the N2a cells to be susceptible to higher concentrations of SPIONs, and consequently, this results in less viability at C4, C5, and C6. Also, the concentration of SPIONs is a critical factor for cell viability with increasing concentration correlated with increased toxicity. Based on our TBDE results, the viability is reduced to 47% and 40% in 200 and 300 µg/mL SPION concentrations, respectively.

Moreover, this investigation reports the effect of SPIONs on the cell viability of N2a cells using impedance spectroscopy, microscopy, and viability test assay. These methods were performed in part in multi-well settings, providing proof of principle that this approach is scalable, with potential for high throughput and high content analysis.

Also, microscopy and impedance spectroscopy methods were used to study the toxicity of SPIONs. The microscopic imaging technology used revealed that at a higher SPION concentration, cell density was compromised. Also, microscopic images showed that attachment and confluence of cells were significantly affected by the presence of SPIONs in the mixture. As per the results shown in this section, the exposure of cells to different concentrations of SPIONs affects the proliferation of cells, so that the maximum proliferation is observed when the concentration of SPIONs is at its minimum (C1). The number of N2A cells normally increases over time; however, the presence of SPIONs around the cells appears to restrict the ability of the cells to multiply.

Based on the results, a correlation between the impedance of sensing electrodes exposed to the cells treated with different SPIONs was demonstrated. Arguably, high-precision toxicity tests require a collection of large numbers of data points from multiple experiments. A high throughput impedance-based cell monitoring platform as reported in this study can be an efficient alternative to more traditional approaches, allowing us to perform a large number of experiments simultaneously with lower sample consumption and in a time effective manner. It was also shown that the variation of impedance is influenced by the concentration of both cells and SPIONs. However, the relationship between the changes of impedance or the related electrical components such as R1, R2, and C depends on various parameters such as the specification of electrodes in addition to other biological factors. The impedance spectroscopy offers great advantages of the label-free and low-cost method for assessment of the effectiveness of SPIONs on cells.

We had demonstrated that high throughput impedance-based label-free platform offers great advantages for studying SPIONs in a cell-based context, opening a window of opportunity to design and test the next generation SPIONs with reduced toxicity for biomedical or medical applications. 

## Figures and Tables

**Table 1:** In-vitro toxicity studies of nanoparticles.

Cell	Type	Coat	Size nm	Qualitative Effects	Characterization	Ref.
^1^R-PC12	MNP	No	10	The increase of MNPs does not affect cell viability. MNPs were attached on the outer membrane of the cell and did not penetrate the cells. No cytotoxic effect up to 0.1 mg/mL but at a high concentration of 0.25 mg/mL, 51% of the PC12 cells remained viable after 72 h	XTT cell viability assay, Imaging, and morphometric analysis, ^2^Elec.	[71]
NR-PC12	MNP	Starch	10	The slight decrease in cell viability after 72 h MNPs concentration increased (80 and 70% viability at 0.02 and 0.1 mg/mL, respectively). At 0.25 mg/mL, MNPs were toxic to PC12 cells. After 24 h no cells remained viable		
R-PC12	MNP	Dextran	10	Cell viability decreased at an MNP concentration of 0.25 mg/mL		
R-PC12	MNP	NO	20	MNPs penetrated the cell without any toxic effect. Morphology patterns of cells are not affected		
^3^L929	SPION	^4^PVA	20–30	17.8% uncoated & 34.6% modified SPIONs viability, Affects viability, Bubble formation	Ultraviolet visible spectroscopy (UV/vis), MTT Assay, Optical Microscopy	[72]
^5^NIH3T3	SPION		10–50	95% of the cells were viable within 3–24 h of incubation and a slight decrease in viability was observed after 48 h of incubation. A slight reduction of viability, Localization of SPIONs in the vesicle, No functionalized SPIONs accumulation in cells, nucleus, and none are toxic at a desirable concentration, negative contrast in the MRI	XTT cell viability assay, bright-field microscopy, MR Imaging	[73]
^6^TK6	Iron oxide U-Fe3O4	No	5–13	U-Fe3O4 NPs did not show a toxic effect, The TBE assay showed slightly reduced cell viability, of TK6 cells at 45 mg/cm^2^ (76% after 0.5 h; 66% after 2 h) whereas 75 mg/cm^2^ strongly decreased cell viability (42.5% after 0.5 h; 48% after 2 h)	Trypan blue exclusion. Relative Growth Activity Assay using Automated Cell Counter (Invitrogen). CBPI and by incorporation of 3H-TdR into DNA of proliferating blood cells. Electron Microscopy	[74]
^6^TK6	iron oxide OC-Fe3O4	Oleate	5–12	OC-Fe3O4 NPs were found to be toxic and affected DNA and morphology of the cells, Viability was reduced to 7.5% for those that were exposed to 30 mg/cm^2^ OC-Fe3O4 NPs		
^7^A3	Iron oxide	^8^Car/A-G	10–50	LC50 of A3 on 1hr-FDA, 24hr FDA, and WST-1 assay, Toxicity vary with the mass concentration, the total number of particles per well, and the total surface area of particles per well	Fluorescein diacetate (FDA) uptake based cytotoxicity assay, WST-1 Assay	[75]
^9^bEnd.3	AmS-IONPs	^10^AminS	27	Toxicity is dependent on surface coating. At concentration above 200 µg/mL reduced neuron viability by 50% in the presence or absence of a magnetic field, 20% reductions in viability were observed with COOH-AmS-IONPs. With an applied magnetic field, AmS-IONPs reduced viability to 75% in astrocyte cultures. COOH-AmS-IONPs caused 65% and 35% viability reduction in the absence and presence of a magnetic field, respectively	MTT Assay, Electron Microscopy	[76]
^11^A549	SPIONs	No	9.3 ± 1.4	Viability Fe_3_O_4_@COOH is greater than 80% at 1000 μg/mL compared to control cells, while bare Fe_3_O_4_ and Fe_3_O_4_@NH2 displayed viability higher than 80% at a concentration of 100 μg/mL and less. No mortality was observed, Decreased cell Proliferation, Effect was dose-dependent	Trypan Blue Dye Exclusion Assay, MTT Assay, Resazurin based PrestoBlue (PB) assay	[77]
^11^A549	SPIONs	@NH2	9 ± 1.3			
^11^A549	SPIONs	@COOH	10.4 ± 1.6			
^12^C17.2/PC12	iron oxide	^13^DexE	14	Endorem uptake = 46.59 ± 4.70 μg Fe/cell.	lactate dehydrogenase assay, CytoTox 96 non-radioactive cytotoxicity assay, manual counting using a Bürker Chamber was used for cell proliferation, No significant changes in cell surface area between control cells and IONP-treated cells could be observed, High intracellular IONP concentrations affect focal adhesions and proliferation, (slows cell cycle progression and decrease proliferation)	[79]
^12^C17.2/PC12	iron oxide	^14^CarXR	14	Resovist uptake = 31.99 ± 2.99 μg Fe/cell.		
^12^C17.2/PC12	iron oxide	lipid-coated ^15^MLs and	14	Cationic MLs = 67.37 ± 5.98 pg Fe/cell		
^12^C17.2/PC12	iron oxide	^16^VSOP	14	VSOPs uptake = 18.65 ± 2.07 pg Fe/cell Control = 100% viability The NPs value being uptaken		
^17^RCGC	MNPs	^18^DMSA	80/120	alter the cell morphology nor compromise cell viability, concentration and time-dependent, DMSA-coated IONPs are not acutely toxic to cultured neurons and that a protein corona around the particles strongly affects their interaction with neurons, cell viability indicated by the low extracellular LDH activity (around 20% of total), while 80% of the LDH remained cellular	lactate dehydrogenase (LDH), MTT assay	[80]
^19^MCF-7	SPIONs	^20^DOX	10 ± 2	DOX-SPION suspension was significantly more active against MCF-7 cells than DOX solution, DOX in solution = 10% mortality, DOX-SPION suspension cell mortality = nearly 40%,	tetrazolium dye (MTT) assay	[81]
^19^MCF-7	SPIONs	^18^DMSA	15	At 24 h MTT Assay ≥ 96% viability about the control, Trypan Blue Assay ≥ 90% cell survival. There was no significant effect on cell morphology, cytoskeleton organization, cell cycle distribution, reactive oxygen species generation, and cell viability compared to the control	MTT Assay, Trypan Blue Assay, Bright field, and fluorescence microscopy	[82]

^1^Rat pheochromocytoma PC12 cells, ^2^Electrophysiological measurements, ^3^L929 mouse fibroblast, ^4^polyvinyl alcohol PVA, ^5^Mouse embryonic fibroblasts NIH3T3, ^6^TK6 human lymphoblast cells, ^7^A3 human T lymphocyte, ^8^Carboxyl/Amine group, ^9^mouse brain-derived microvessel endothelial cell line, bEnd.3, ^10^Aminosil, ^11^A549 human lung epithelial cancer cells, ^12^C17.2 neural progenitor cells, and PC12 rat pheochromocytoma cells, ^13^dextran-coated Endorem, ^14^carboxydextran-coated Resovist, ^15^magnetoliposomes, ^16^citrate-coated very small iron oxide particles, ^17^RCGC primary rat cerebellar granule cells/neurons, ^18^dimercaptosuccinic acid, ^19^Human breast cancer MCF-7 cell, ^20^doxorubicin.

**Table 2:** Comparative use of impedance-based for cellular analysis.

Cells/tissue	Types of Impedance-Based Assay	Cellular Analysis	Ref.
^1^S1barrel cortex	Impedance spectra using HP4284 LCR meter with Implanted electrodes	Identify changes of impedance magnitude at 1kHz. Results suggested that change in impedance is due to the distribution and reactions of cells around the implanted electrodes.	[95]
^2^MVEC	^7^ECIS	Quantify cell behavior such as adhesion, proliferation, cell migration, formation, and maturation of a confluent cell barrier, and wound healing after the application of an electrical wound	[96]
^3^OSCC	ECIS	Monitor cell adhesion, spreading, proliferation and apoptosis after the addition of anti-cancer drug-cisplatin.	[97]
^4^MBMEC	Impedance spectroscopy using cellZscope	Investigate the integrity and permeability of endothelial cells.	[100]
^5^U87MG	Single-cell bioelectrical impedance using single and multi-cell electrodes	Monitor change in shape and impedance after introducing chlorotoxin, an ion channel inhibitor.	[98]
^6^hESC-CMs	Cardiomyocytes Impedance Assay using gold film electrodes and MEA	Detection of beating and toxicity of drugs to cardiomyocytes	[99]

^1^S1, primary somatosensory barrel cortex, ^2^microvascular endothelial cells, ^3^oral squamous cell carcinoma, ^4^mouse brain microvascular Endothelial Cells, ^5^Human glioblastoma cells, ^6^human embryonic stem cell-derived cardiomyocytes, ^7^Electric cell-substrate impedance sensing.

**Table 3:** Impedance measurement in a range of frequencies (f_1_–f_N_) at different times (T1–T8).

f	T1	T2	…	T8	Z_MAX_(f)	Z_MIN_(f)	Z_MAX_−Z_MIN_(f)
f_1_	Z_0_(f_1_)	Z_1_(f_1_)	…	Z_72_(f_1_)	Max (Z_0_(f_1_) … Z_72_(f_1_))	Min (Z_0_(f_1_) … Z_72_(f_1_))	Z_Max_−Z_MIN_(f_1_)
f_2_	Z_0_(f_2_)	Z_1_(f_2_)	…	Z_72_(f_2_)	Max (Z_0_(f_2_) … Z_72_(f_2_))	Min (Z_0_(f_1_) … Z_72_(f_1_))	Z_Max_−Z_MIN_(f_2_)
…	…	…	…	…	…	…	…
f_N_	Z_0_(f_N_)	Z_1_(f_N_)	…	Z_72_(f_N_)	Max (Z_0_(f_N_) … Z_72_(f_N_))	Min (Z_0_(f_1_) … Z_72_(f_1_))	Z_maz_−Z_MIN_(f_N_)

**Table 4:** Electric Equivalent Circuit for each range of frequencies.

f	T1	T2	…	T8
f_1_	Z_0_(f_1_)	Z_1_(f_1_)	…	Z_72_(f_1_)
f_2_	Z_0_(f_2_)	Z_1_(f_2_)	…	Z_72_(f_2_)
…	…	…	…	…
f_N_	Z_0_(f_N_)	Z_1_(f_N_)	…	Z_72_(f_N_)
f1–f_N_	C_0_, R_1,0_, R_2,0_	C_1,1_, R_1,1_, R_2,1_	…	C _72_, R_1,72_, R_2,72_

**Table 5:** Continuation of Table 1, AVG and STD analysis.

F	AVG (f)	STD(f)	Z_MAX_−Z_MIN_(f)/AVG(f)
f_1_	AVG (Z_0_(f_1_) … Z_72_(f_1_))	STD (Z_0_(f_1_) … Z_72_(f_1_))	Z_Max_−Z_MIN_(f_1_)/AVG(f_1_)
f_2_	AVG (Z_0_(f_2_) … Z_72_(f_2_))	STD (Z_0_(f_1_) … Z_72_(f_1_))	Z_Max_−Z_MIN_(f_2_)/AVG(f_2_)
…	…	…	…
f_N_	AVG (Z_0_(f_N_) … Z_72_(f_N_))	STD (Z_0_(f_1_) … Z_72_(f_N_))	Z_maz_−Z_MIN_(f_N_))/AVG(f_N_)

**Table 6:** A sample of impedance measurement in 8 different times, in the range of 0.1–100 KHz, when the concentration of SPIONs is C1.

Frequency	T1	T2	T3	T4	T5	T6	T7	T8
0.1	1,591,311	1,788,090	1,845,433	1,905,271	1,936,466	2,551,464	2,646,480	2,483,508
0.12589	1,392,229	1,613,690	1,654,271	1,705,281	1,740,582	2,358,748	2,393,614	2,261,709
0.15849	1,229,815	1,444,350	1,489,574	1,552,677	1,580,039	2,194,239	2,242,218	2,077,649
0.19953	1,100,665	1,306,554	1,349,952	1,402,120	1,420,733	2,052,875	2,064,253	1,917,803
0.25119	986,997.7	1,195,129	1,236,538	1,279,733	1,300,714	1,897,634	1,919,006	1,770,034
0.31623	895,652.1	1,091,249	1,135,028	1,172,615	1,195,926	1,761,568	1,799,637	1,629,005
0.39811	822,172.1	1,018,880	1,057,300	1,087,263	1,113,011	1,680,547	1,677,912	1,525,727
0.50119	751,371.5	952,111	990,455.9	1,019,149	1,046,336	1,594,401	1,576,466	1,419,292
0.63096	712,562.2	900,056	938,485.5	977,048.7	990,141.5	1,478,979	1,491,643	1,323,400
0.79433	664,134.2	856,963.1	892,184.2	915,849.7	946,656.1	1,457,470	1,394,427	1,232,235
1	627,103.2	818,777.4	853,829.3	883,702.3	904,551.5	1,357,505	1,305,211	1,147,258
…	…	…	…	…	…	…	…	…
25,119	2644.421	2698.195	2725.695	2741.836	2736.834	3013.611	3282.717	3493.791
31,623	2424.111	2486.919	2519.135	2537.534	2534.271	2838.178	3113.052	3330.472
39,811	2247.619	2316.085	2352.01	2371.349	2370.603	2688.499	2965.835	3184.751
50,119	2103.743	2173.723	2209.435	2232.546	2232.609	2555.514	2826.027	3048.819
63,096	1984.099	2052.586	2089.326	2112.155	2113.513	2429.64	2689.563	2913.769
79,433	1881.111	1947.183	1981.887	2004.221	2006.622	2307.667	2551.561	2773.715
100,000	1789.252	1848.986	1882.076	1903.488	1906.164	2183.312	2401.9	2619.237
